# The marine‐derived furanone reduces intracellular lipid accumulation in vitro by targeting LXRα and PPARα

**DOI:** 10.1111/jcmm.15012

**Published:** 2020-01-24

**Authors:** Ting Li, Shu‐Mei Hu, Xiao‐Yan Pang, Jun‐feng Wang, Jia‐Yu Yin, Fa‐Hui Li, Jin Wang, Xiao‐Qian Yang, Bin Xia, Yong‐Hong Liu, Wei‐Guo Song, Shou‐Dong Guo

**Affiliations:** ^1^ Institute of Lipid Metabolism and Atherosclerosis School of Pharmacy Innovative Drug Research Centre Weifang Medical University Weifang China; ^2^ CAS Key Laboratory of Tropical Marine Bio‐resources and Ecology/Guangdong Key Laboratory of Marine Materia Medica/RNAM Center for Marine Microbiology South China Sea Institute of Oceanology Chinese Academy of Sciences Guangzhou China

**Keywords:** acetyl‐CoA carboxylase, fatty acid synthase, furanone, LXR antagonist, PPAR antagonist, PPARα agonist, reverse cholesterol transport

## Abstract

Recent studies have demonstrated that commercially available lipid‐lowering drugs cause various side effects; therefore, searching for anti‐hyperlipidaemic compounds with lower toxicity is a research hotspot. This study was designed to investigate whether the marine‐derived compound, 5‐hydroxy‐3‐methoxy‐5‐methyl‐4‐butylfuran‐2(5H)‐one, has an anti‐hyperlipidaemic activity, and the potential underlying mechanism in vitro. Results showed that the furanone had weaker cytotoxicity compared to positive control drugs. In RAW 264.7 cells, the furanone significantly lowered ox‐LDL‐induced lipid accumulation (~50%), and its triglyceride (TG)‐lowering effect was greater than that of liver X receptor (LXR) agonist T0901317. In addition, it significantly elevated the protein levels of peroxisome proliferator‐activated receptors (PPARα) and ATP‐binding cassette (ABC) transporters, which could be partially inhibited by LXR antagonists, GSK2033 and SR9243. In HepG2 cells, it significantly decreased oleic acid‐induced lipid accumulation, enhanced the protein levels of low‐density lipoprotein receptor (LDLR), ABCG5, ABCG8 and PPARα, and reduced the expression of sterol regulatory element‐binding protein 2 (~32%). PPARα antagonists, GW6471 and MK886, could significantly inhibit the furanone‐induced lipid‐lowering effect. Furthermore, the furanone showed a significantly lower activity on the activation of the expression of lipogenic genes compared to T0901317. Taken together, the furanone exhibited a weak cytotoxicity but had powerful TC‐ and TG‐lowering effects most likely through targeting LXRα and PPARα, respectively. These findings indicate that the furanone has a potential application for the treatment of dyslipidaemia.

## 1 INTRODUCTION

Hyperlipidaemia is an important atherogenic factor by promoting the formation and accumulation of lipid plaques in the arteries.^1^, ^2^ Currently, statins are the leading lipid‐lowering drugs that inhibit 3‐hydroxy‐3‐methylglutaryl coenzyme A (HMG‐CoA) reductase, the rate‐limiting enzyme for de novo synthesis of cholesterol.^3^ However, increasing numbers of studies have reported side effects of statins.^4^, ^5^ As reviewed by Watts and Eckel, discontinuation is a key problem for statins. This can be mainly attributed to the development of statin‐associated muscle symptoms and new‐onset type 2 diabetes mellitus; the skeletal muscle symptoms may arise from the decreased function of the chloride channel member 1 in statin treatment patients.^6^, ^7^ Other side effects of statins include potential adverse neurological and neurocognitive effects, hepatotoxicity and renal toxicity.^4^

Peroxisome proliferator‐activated receptors (PPARs) including three sub‐family members, PPARα, PPARβ/δ and PPARγ, are nuclear receptors that regulate lipid metabolism via their transcriptional activity. They activate the expression of target genes by binding to specific repeat DNA response elements with their obligate heterodimeric partner retinoid X receptor (RXR).^8^ PPARα modulates the transcription of genes encoding key enzymes in fatty acid catabolism pathway such as carnitine palmitoyltransferase 1A and 2, acyl‐CoA dehydrogenase and 3‐hydroxy‐3‐methylglutaryl‐CoA synthase 2.^9^ PPARs are activated by fatty acids and eicosanoids, and inactivated by synthetic compounds such as GW6471 and MK886. PPARα agonists such as fibrates can lower TG and increase the levels of high‐density lipoprotein cholesterol (HDL‐C).^10^, ^11^ Thus, fibrates play key roles in the treatment of lipid disorders.^12^ However, they also exhibit various side effects, for example, rhabdomyolysis, liver toxicity and nephrotoxicity.^13^, ^14^, ^15^

Liver X receptors (LXRs) are another kind of nuclear receptor that plays key roles in the regulation of lipid metabolism. LXRs are composed of two isotypes, LXRα and LXRβ, all of them can form obligate heterodimers with RXRα and then bind to a specific DNA recognition sequence known as an LXR response element.^16^, ^17^ The LXRs are activated by endogenous ligands such as oxysterols, intermediate precursors in the cholesterol biosynthetic pathway such as desmosterol, and synthetic agonists such as T0901317. They are inactivated by inhibitors such as GSK2033 and SR9243.^16^ Upon activation, LXRs improve the initial step of reverse cholesterol transport (RCT) by up‐regulation of its target genes ATP‐binding cassette (ABC) G1 and ABCA1 in peripheral cells such as macrophages. In hepatocytes, LXRα stimulates the expression of cholesterol 7 alpha‐hydroxylase A1 (CYP7A1), a rate‐limiting enzyme in the bile acid synthesis pathway, and up‐regulates the biliary cholesterol excretion via directly activating the expression of ABCG5 and G8 in the liver.^16^ Furthermore, LXRs accelerate cholesterol excretion in the small intestine via activation of the expression of ABCG5 and G8. However, a big problem for LXRs agonists is that they can accelerate lipogenesis in the liver mainly due to the transcriptional induction of lipogenic genes such as sterol regulatory element‐binding protein (SREBP)‐1c, fatty acid synthase (FAS), acetyl‐CoA carboxylase 1 (ACC1), stearoyl‐CoA desaturase 1 (SCD1) and diacylglycerol O‐acyltransferase (DGAT), increasing hepatic TG levels.^16^, ^17^, ^18^

Furanone, a five‐membered heteroaromatic ring containing an oxygen atom, is considered as one of the pharmacophores of biologically active substances. Furanones are classified into three main types: 2(*3H*)‐furanones, 2(*5H*)‐furanones and 3(*2H*)‐furanones. Furanone is present in lots of natural products including food and has been reported to have various biological functions such as anticancer, antiviral, antifungal, antibacterial, anti‐inflammatory, antioxidant, antiarthritic and anti‐hyperlipidaemic.^19^ The best known and most studied furanone is ascorbic acid (Vitamin C). During the past decades, researchers paid a great deal of attention to the synthesis of furanone derivatives and investigation of their pharmacological activities.^19^, ^20^ Husain et al reviewed therapeutic potential of furanone derivatives in the literature from 1987 to 2018, focusing on their anti‐inflammatory, anticancer and antimicrobial activities.^19^ We have reported 14 new and 17 known metabolites from the fungus *Setosphaeria* sp SCSIO41009.^21^ Here, we reported for the first time that the furanone named as 5‐hydroxy‐3‐methoxy‐5‐methyl‐4‐butylfuran‐2(5H)‐one had an effective lipid‐lowering activity via influencing multiple processes of lipid metabolism.

## 2 MATERIALS AND METHODS

### 2.1 Materials

Mouse‐derived macrophage cell line RAW 264.7 and the human hepatoma cell line HepG2 were purchased from the Cell Bank of Chinese Academy of Sciences. (Shanghai, China). 3‐(4,5‐dimethyl‐2‐thiazolyl)‐2,5‐diphenyl‐2H‐tetrazolium bromide (MTT, 413Y0511), oleic acid (01008) and Oil Red O (00625) were Sigma‐Aldrich products (St. Louis, MO, USA). Liver X receptor (LXR) agonist T0901317 (293754‐55‐9), fenofibrate (S1794) and the peroxisome proliferator‐activated receptor (PPAR) α antagonist MK886 were the products of Selleck (Shanghai, China). LXR antagonist, GSK2033 and SR9243, and PPARα antagonist GW6471 were the products of MedChemExpress (Shanghai, China). Dimethyl sulphoxide (DMSO, 821D035) and the goat serum (SL038) were purchased from Solarbio (Beijing, China). Dulbecco's modified Eagle's medium (DMEM) and foetal bovine serum (FBS) were from Gibco (BRL, Gaithersburg, MD, USA). RIPA lysis buffer was a product of Merck (3108491; Darmstadt, Germany). Rabbit polyclonal antibody against LXRα (ab3585, 1:200; ab176323, 1:5000) and LXRβ (ab28479, 1:500); rabbit monoclonal antibody against scavenger receptor B type 1 (SR‐B1, ab217318, 1:2000), ATP‐binding cassette (ABC) G1 (ab52617, 1:1000) and low‐density lipoprotein receptor (ab52818, LDLR 1:1000); and mouse monoclonal antibody against ABCA1 (ab18180, 1:200 or 1:1000) were from Abcam (Cambridge, MA, USA). Mouse monoclonal antibody against sterol regulatory element‐binding protein (SREBP)‐1c (sc‐13551, 1:100), SREBP‐2 (sc‐271616, 1:200) and PPARα (sc‐398394, 1:100) were purchased from Santa Cruz Biotechnology (Santa Cruz, CA, USA). Rabbit polyclonal antibody against cholesterol 7 alpha‐hydroxylase A1 (CYP7A1, TA351400, 1:1000) was the product of OriGene (Shanghai, China). Mouse monoclonal antibody against β‐actin (66009‐1‐Ig, 1:5000), rabbit polyclonal antibody against ABCG5 (27722‐1‐AP, 1:1000) and rabbit monoclonal antibody against proprotein convertase subtilisin/kexin type 9 (PCSK9, 55206‐1‐AP, 1:500) were the products of Proteintech (Chicago, IL, USA). Complete protease inhibitor and the secondary antibodies, including the goat antimouse IgG (FITC conjugated), were from CWBIO (Beijing, China). Mouse monoclonal antibody against ABCG8 (1B10A5, 1:1000) and enhanced chemiluminescence (ECL) kits were purchased from Thermo Scientific Pierce (Rockford, IL, USA). Total cholesterol (TC) and triglyceride (TG) assay kits were the products of Biosino Bio‐technology and Science Inc (Beijing, China). Double‐deionized water was produced using a Milli‐Q Gradient System from Millipore. All reagents used in this study were of analytical grade.

### 2.2 Purity determination of the furanone, 5‐hydroxy‐3‐methoxy‐5‐methyl‐4‐butylfuran‐2(5H)‐one

The furanone, 5‐hydroxy‐3‐methoxy‐5‐methyl‐4‐butylfuran‐2(5H)‐one, was isolated from the fungus *Setosphaeria* sp SCSIO41009, as previously reported.^21^ Its purity was determined by multiple analytical methods. Ultra‐performance liquid chromatography (UPLC) spectrum was performed on an Acquity UPLC BEH C18 column (2.1 × 50 mm i.d., 1.7 μm) connected to a Waters Acquity H Class UPLC System (Waters) with a PDA detector (wavelength of 212 nm). High‐resolution electrospray ionization mass spectrometry (HRESIMS) spectrum was recorded on a Bruker maXis Q‐TOF mass spectrometer in positive ion mode. 1D and 2D NMR spectra were measured on a Bruker AV 500 MHz or AVANCE HD 700 MHz NMR spectrometer with tetramethylsilane as an internal standard.^21^

### 2.3 Preparation of lipoproteins

Plasma was obtained from healthy volunteers at the Affiliated Hospital of Weifang Medical University. To obtain LDL fraction, plasma was subjected to sequential ultracentrifugation as previously described.^22^, ^23^ In brief, the plasma density was adjusted to 1.006 g/mL for ultracentrifugation at 10°C (400 000 × *g* for 24 hours). The upper layer containing very low‐density lipoproteins was removed, and the density was re‐adjusted to 1.063 g/mL for ultracentrifugation at 400,000 × g for an additional 24 hours to obtain the upper layer containing low‐density lipoproteins (LDL). EDTA‐2Na (0.1%, w/v) was added to chelate the metal ions, thereby reducing oxidation during ultracentrifugation. The protein content of the fractions was determined by the Bradford method. The LDL fraction was stored at 4°C until use.

Oxidized LDL (Ox‐LDL) was prepared by the method described previously.^23^ In brief, LDL (~10 mg/mL) was incubated with CuSO_4_ (10 μmol/L) at 37°C for 24 hours; then, the reaction was stopped by addition of 500 μmol/L EDTA‐2Na. The resulting ox‐LDL was dialysed against 0.01 M phosphate‐buffered saline (PBS, PH = 7.4) at 4°C for 24 hours and then filtered through a 0.22‐μm filter and stored at 4°C until use.

### 2.4 Cell culture

RAW 264.7 macrophages or HepG2 cells were seeded in a 25‐cm^2^ flask and then cultured in DMEM supplemented with 10% FBS, 100 U/mL penicillin and 100 μg/mL streptomycin. Cells were grown in a humidified 5% CO_2_ incubator at 37°C. All compounds used were dissolved in DMSO. The final concentration of DMSO in cell culture was 0.1%. Controls groups were treated with the vehicle (0.1% DMSO) alone.^24^

### 2.5 Cell viability assay

RAW 264.7 or HepG2 cells were seeded in a 96‐well plate at a density of 1.0 × 10^4^ cells per well. Cells were treated with 0, 1.0, 2.5, 5 and 10 µmol/L of the furanone, fenofibrate or T0901317 for 24 hours. Cell viability was evaluated by the MTT method.^24^, ^25^ In brief, after the treatment period, 20µL of 5.0 mg/mL MTT was added to each well. Cells were then incubated for an additional 2 hours for the formation of formazan crystals. After washing cells in PBS for 3 times, formazan was solubilized in 150 µL of DMSO, and the optical density at 570 nm was recorded using a SpectraMax i3x Multi‐Mode Microplate Platform (Molecular Devices, San Jose, CA, USA). Controls were defined as groups with vehicle only treatment.

### 2.6 Oil Red O staining

For Oil Red O staining, cells grown on glass coverslips in six‐well plates were fixed with 4% (w/v) paraformaldehyde at room temperature for 30 minutes and then stained with filtered Oil Red O solution (5 mg/mL in 60% isopropanol) at room temperature for 1 hour. Afterwards, the coverslips were washed with H_2_O for several times, dried and mounted on slides.^26^, ^27^ Lipid‐stained area was captured and quantified using Axio Vert.A1 inverted microscope (Zeiss, Jena, Germany), and images were recorded with an Axiocam 506 colour camera (Zeiss).

### 2.7 Measurement of intracellular lipid levels

For RAW264.7 macrophages, cells were seeded in six‐well plates and incubated with ox‐LDL (50 μg/mL) in DMEM without FBS for 24 hours. Afterwards, cells were treated with 5 µmol/L of the furanone, 1 µmol/L of T0901317 or 5 µmol/L of fenofibrate dissolved in DMEM supplemented with 10% FBS for an additional 24 hours. For the LXR inhibition experiments, 10 µmol/L of LXR antagonist GSK2033 or SR9243 was added 2 hours before the addition of the furanone.^27^, ^28^ The cells were washed in PBS for 3 times and treated with 0.2 mL of RIPA lysis buffer at 4°C for 30 minutes.^29^, ^30^ The obtained mixture was heated at 70°C for 10 minutes in a water bath and then centrifugated at 1500 × *g* for 5 minutes. The obtained supernatant was used to detect the lipid levels by assay kits according to the manufacturer's instructions. The absorbance at 505 nm was recorded by a SpectraMax i3x Multi‐Mode Microplate Platform (Molecular Devices, San Jose, CA, USA).

HepG2 cells were incubated with 0.5 mmol/L oleic acid for 24 hours. Then, cells were washed with PBS for 3 times and treated with 5 µmol/L of the furanone, 1 µmol/L of T0901317 or 5 µmol/L of fenofibrate for an additional 24 hours. For PPARα inhibition experiments, 10 µmol/L of PPARα antagonist MK886 or GW6471 was added 2 hours before the addition of the furanone.^27^, ^28^ Afterwards, lipids in cells were extracted and measured using the same methods as described above. The same treatment was also used to investigate the changes in the levels of mRNA and protein.

### 2.8 Fluorescent immunocytochemistry assay

The treated RAW 264.7 macrophages grown on glass coverslips were washed with PBS 3 times, fixed with 4% paraformaldehyde, permeabilized with 0.1% Triton X‐100 and blocked in 3% BSA. After blocking in goat serum for 2 hours at room temperature, the cells were incubated with a primary antibody (1:200) in PBS containing 0.1% Tween‐20 (PBS‐T) overnight at 4°C. After washing 3‐5 times in PBS‐T, the coverslips were incubated with a goat antimouse IgG (FITC conjugated, 1:100 in PBS‐T) and 4’,6‐diamidino‐2‐phenylindole (DAPI). The coverslips were then washed in PBS and mounted on slides.^23^, ^26^ Images were captured using Axio Vert.A1 inverted microscope (Zeiss).

### 2.9 Real‐time quantitative PCR

Total RNAs were isolated from cells using TRIzol reagent (SparkJade, Qingdao, China) according to the manufacturer's instructions. The concentration and purity of total RNAs were determined spectrophotometrically by measuring the absorbance at 260 nm and 280 nm using a UV spectrophotometer, and cDNA was produced using an ABI Veriti 96‐Well Thermal Cycler (Waltham, MA, USA) and FastQuant RT Kit with gDNase (Tiangen). Real‐time PCR was performed in an ABI QuantStudio3 PCR System (Waltham, MA, USA) using SYBR Green qPCR Master Mix and gene‐specific primers with an initial denaturation step at 95°C for 10 minutes followed by 40 cycles of 95°C for 15 seconds, 58°C for 30 seconds and 68°C for 60 seconds. The primers for qRT‐PCR are listed in Table 1. The fold change in the expression of targets relative to the housekeeping gene *GAPDH* was calculated based on the 2^‐ΔΔCt^ relative expression formula.

**Table 1:** The primers used for the polymerase chain reaction (PCR) reaction

Primer	Sequences (5′–3′)
mGAPDH	
Forward	AGGTCGGTGTGAACGGATTTG
Reverse	GGGGTCGTTGATGGCAACA
mABCA1	
Forward	GTTACGGCAGATCAAGCATCC
Reverse	TGGAAGGGACAAATTGTGCTG
mABCG1	
Forward	GCTCCATCGTCTGTACCATCC
Reverse	ACGCATTGTCCTTGACTTAGG
mLXRα	
Forward	CTCAATGCCTGATGTTTCTCCT
Reverse	TCCAACCCTATCCCTAAAGCAA
mLXRβ	
Forward	ATGTCTTCCCCCACAAGTTCT
Reverse	GACCACGATGTAGGCAGAGC
mABCG5	
Forward	AGAGGGCCTCACATCAACAGA
Reverse	CTGACGCTGTAGGACACATGC
mPPARα	
Forward	AACATCGAGTGTCGAATATGTGG
Reverse	CCGAATAGTTCGCCGAAAGAA
mFAS	
Forward	CATCCACTCAGGTTCAGGTG
Reverse	AGGTATGCTCGCTTCTCTGC
mACC1	
Forward	GAGGTACCGAAGTGGCATCC
Reverse	GTGACCTGAGCGTGGGAGAA
mDGAT1	
Forward	TGGTGTGTGGTGATGCTGATC
Reverse	GCCAGGCGCTTCTCAA
mDGAT2	
Forward	GGCTACGTTGGCTGGTAACT
Reverse	CACTCCCATTCTTGGAGAGC
mSCD1	
Forward	CATCATTCTCATGGTCCTGCT
Reverse	CCCAGTCGTACACGTCATTTT
mSREBP‐1c	
Forward	TGGACGAGCTGGCCTTCGGT
Reverse	GGCCAGCGGCAGGCTAGATG
hGAPDH	
Forward	ACAACTTTGGTATCGTGGAAGG
Reverse	GCCATCACGCCACAGTTTC
hSR‐B1	
Forward	AATAAGCCCATGACCCTGAAGC
Reverse	GCCCCACATGATCTCACCC
hPPARα	
Forward	ATGGTGGACACGGAAAGCC
Reverse	CGATGGATTGCGAAATCTCTTGG
hCYP7A1	
Forward	GAGAAGGCAAACGGGTGAAC
Reverse	GGATTGGCACCAAATTGCAGA
hLDLR	
Forward	TCTGCAACATGGCTAGAGACT
Reverse	TCCAAGCATTCGTTGGTCCC
hSREBP‐2	
Forward	GGTCATTCACCCAGGTCACA
Reverse	TACCTGGGAGGATGTCACCA
hSREBP‐1c	
Forward	GACAGCCCAGTCTTTGAGGA
Reverse	GAGAAGCACCAAGGAGACGA
hFAS	
Forward	CCATCTACAACATCGACACCAG
Reverse	CTTCCACACTATGCTCAGGTAG
hACC1	
Forward	TACCTTCTTCTACTGGCGGCTGAG
Reverse	GCCTTCACTGTTCCTTCCACTTCC
hDGAT1	
Forward	CTTTTTCCAGGGCAACTATGG
Reverse	ATAGTTGAGCACGTAGTAGTCG
hDGAT2	
Forward	TTTCGAGACTACTTTCCCATCC
Reverse	GAACTTCTTGCTCACTTCTGTG
hSCD1	
Forward	CCGACGTGGCTTTTTCTTCT
Reverse	GCGTACTCCCCTTCTCTTTGAC
hABCG5	
Forward	ACTGCTTCTCCTACGTCCTG
Reverse	CTGTAGTTGCCAATCAGTCGG
hABCG8	
Forward	CTGTGGAATGGGACTGTACTTC
Reverse	GTTGGACTGACCACTGTAGGT

### 2.10 Protein isolation, electrophoresis and Western blotting

Total proteins from the cells were extracted using RIPA lysis buffer with complete protease inhibitor according to the manufacturer's instructions. Equal amounts of protein were subjected to 6% or 10% SDS‐PAGE and transferred onto polyvinylidene fluoride membranes by electroblotting. After blocking in Tris‐buffered saline containing 0.1% Tween‐20 and 5% non‐fat dry milk for 2 hours at room temperature, the membranes were incubated with primary antibodies overnight at 4°C. After washing 3 times, the membranes were incubated with horseradish peroxidase‐conjugated secondary antibodies for 2 hours at room temperature. Immunoblots were revealed by enhanced chemiluminescence reaction and visualized using a high‐performance chemiluminescence film. Images were captured by Clinx ChemiScope 6000 Pro (Shanghai, China), and densitometry analysis was conducted using Clinx Image Analysis Software (Shanghai, China). The expression of the proteins was normalized by housekeeping protein β‐actin.^24^, ^31^

### 2.11 Data analysis

All the bioassay results were expressed as the mean ± standard deviation (SD) for at least three independent experiments. Statistical analysis was performed with one‐way analysis of variance (ANOVA) followed by Tukey's test. Differences were considered to be significant at a *P* < .05.

## 3 RESULTS

### 3.1 The purity of the furanone is suitable for pharmacological study

**Figure 1:**
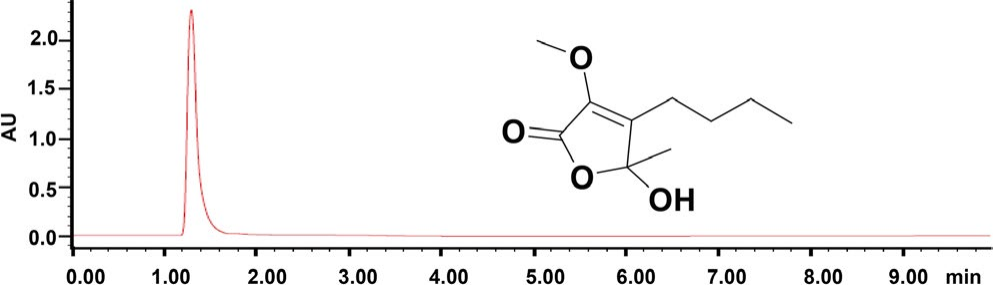
Purity and structure of the furanone, 5‐hydroxy‐3‐methoxy‐5‐methyl‐4‐butylfuran‐2(5H)‐one. Purity assay was carried out using a Waters Acquity H Class UPLC system with a PDA detector

The furanone, 5‐hydroxy‐3‐methoxy‐5‐methyl‐4‐butylfuran‐2(5H)‐one, isolated from the fermentation broth of the fungus *Setosphaeria* sp SCSIO41009 exhibited a single peak on an Acquity UPLC BEH C18 column, and its purity was 98.1% as evaluated by the peak area (Figure 1). This furanone showed a sodium adduct ion at *m/z* 223.0946 [M + Na]^+^, and its molecular formula was established as C_10_H_16_O_4_ by the NMR data.^21^ In general, the purity of a compound above 95% is suitable for pharmacological study.^32^, ^33^ Taken together, these data demonstrated that the furanone is pure enough for further pharmacological study. The detailed structure of the furanone as shown in Figure 1 was described in our previous publication.^21^

### 3.2 The furanone lowered lipid levels in ox‐LDL‐laden RAW264.7 cells

**Figure 2:**
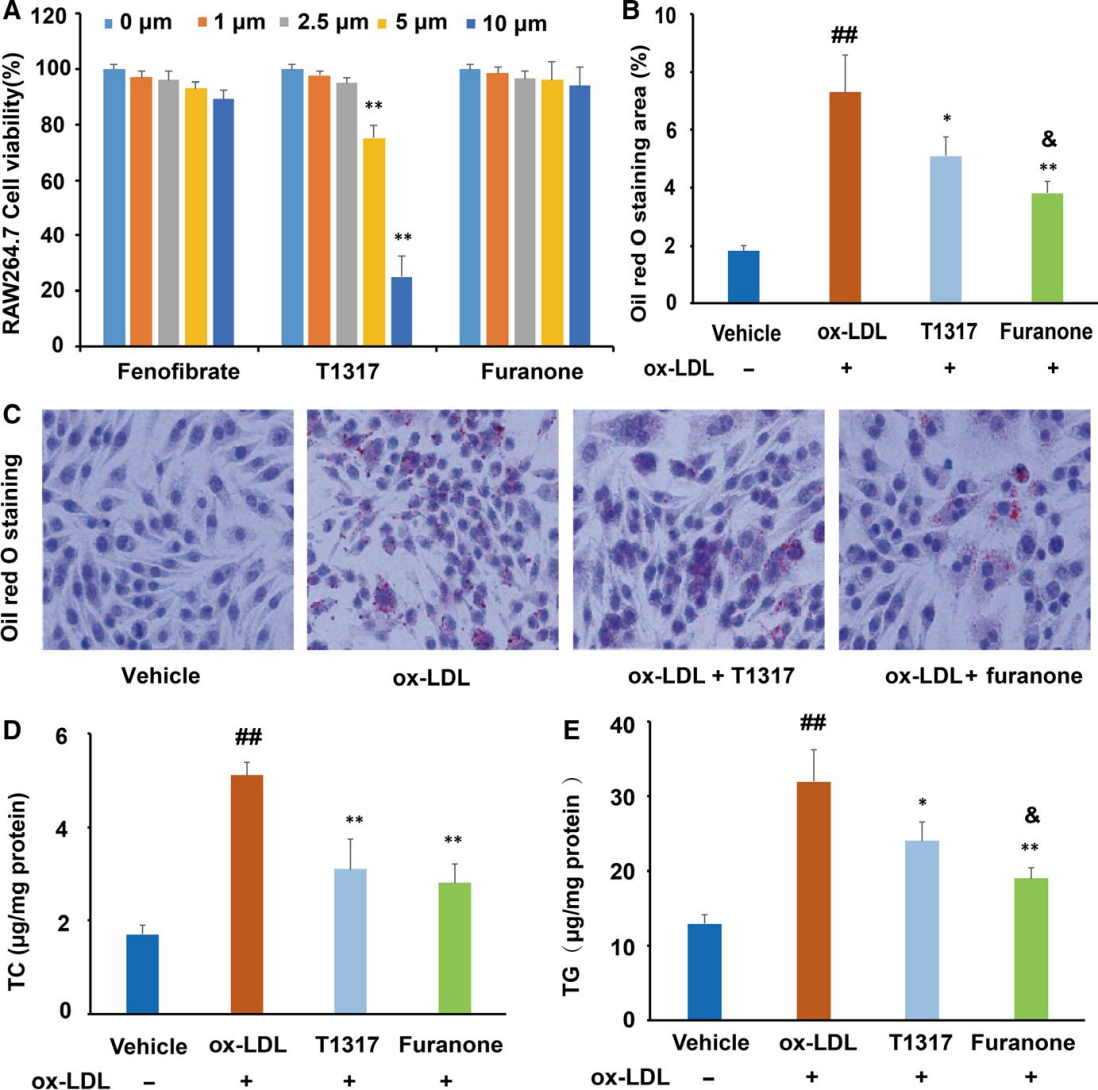
Cytotoxicity and lipid‐lowering activity of the furanone in RAW264.7 cells. (A) Cell viability of RAW264.7 macrophages in the presence of 0‐10 μmol/L furanone, fenofibrate or liver X receptor agonist T0901317; (B) percentage of Oil Red O staining area; (C) typical pictures of Oil Red O staining (100×); (D) intracellular levels of total cholesterol (TC); (E) intracellular levels of triglyceride (TG); T1317: liver X receptor agonist T0901317. Data are expressed as mean ± SD (n = 3). ^##^means *P* < .01 vs vehicle; *means *P* < .05 vs model group; **means *P* < .01 vs model group; ^&^means *P* < .05 vs positive control T1317

The cytotoxicity of the furanone, fenofibrate and LXR agonist T0901317 was determined by the MTT method. As shown in Figure 2A, the furanone and fenofibrate did not show significant cytotoxicity within the concentration up to 10 μmol/L. However, T0901317 reduced cell viability by ~25% and 74% at the concentration of 5 μmol/L and 10 μmol/L, respectively. To avoid cytotoxicity, the concentration of the furanone and fenofibrate was set to 5 μmol/L, and the concentration of LXR agonist T0901317 was set to 1 μmol/L as described previously.^34^

Oil Red O staining results indicated that ox‐LDL at a concentration of 50 μg/mL can significantly elevate the lipid levels in RAW264.7 cells (Figure 2B and C). T0901317 and furanone significantly decreased Oil Red O staining area by ~ 32% (*P* < .05, Figure 2B) and ~48% (*P* < .01, Figure 2B), respectively. Further, TC and TG analysis by assay kits showed that T0901317 markedly reduced the cellular levels of TC (~43%, *P* < .01, Figure 2D) and TG (~33%, *P* < .05, Figure 2E) compared to the model group. The furanone treatment reduced cellular TC and TG levels by ~49% (*P* < .01) and ~58% (*P* < .01), respectively. More importantly, the furanone's TG reduction effect was better than T0901317 (Figure 2E, *P* < .05).

### 3.3 The furanone improved the expression of transporters in RAW264.7 cells

**Figure 3:**
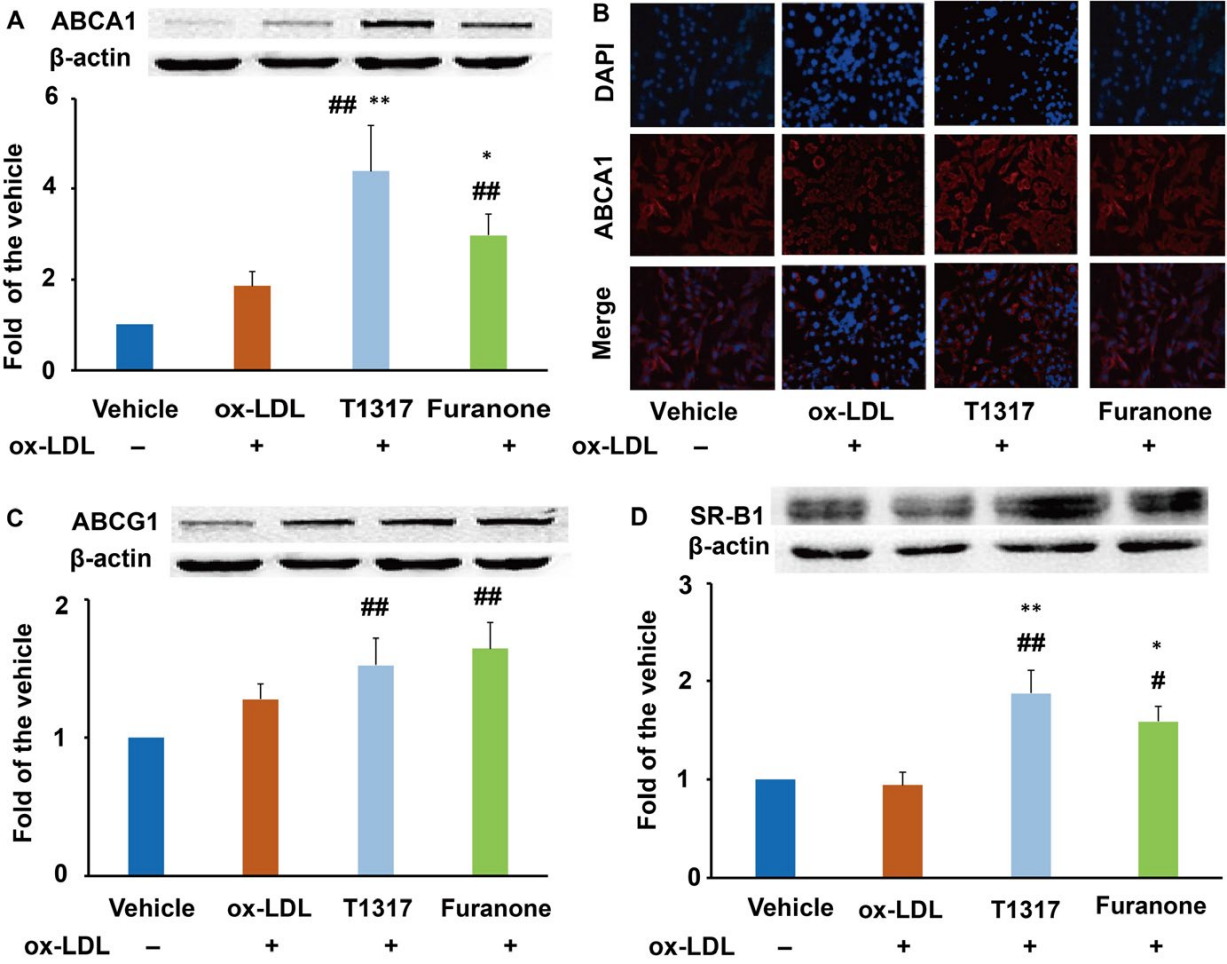
Effect of the furanone on enhancing the protein expression of ABCA1, ABCG1 and SR‐B1 in RAW264.7 cells. (A) Protein expression of ABCA1 and densitometric quantification; (B) detection of ABCA1 expression by fluorescent immunocytochemistry (100×); (C) protein expression of ABCG1 and densitometric quantification; (D) protein expression of SR‐B1 and densitometric quantification. Data are expressed as mean ± SD (n = 4). ^#^means *P* < .05 vs vehicle; ^##^means *P* < .01 vs vehicle; *means *P* < .05 vs model group; **means *P* < .01 vs model group

It is well documented that ABCA1 mediates cholesterol efflux from peripheral cells to apolipoprotein A1 or pre‐β HDL, while ABCG1 and SR‐B1 mediate cholesterol efflux from peripheral cells to mature HDL.^22^ In this study, LXR agonist T0901317 significantly enhanced the protein levels of ABCA1, ABCG1 and SR‐B1 (Figure 3A‐D). It was of note that the furanone increased the expression of ABCA1 about threefold and 60% as compared to the vehicle and model, respectively, as determined by Western blot (Figure 3A) and immunocytochemistry (Figure 3B). Furthermore, the furanone increased the protein levels of ABCG1 and SR‐B1 by ~1.8‐fold and 1.5‐fold, respectively, compared with vehicle group (Figure 3C and D, *P* < .01). However, the effect of T0901317 and furanone on the expression of ABCG1 did not show any significant difference compared to the model group (Figure 3C). Taken together, the effects of this furanone at the concentration of 5 μmol/L on enhancing cholesterol transporters were close to that of LXR agonist T0901317 at the concentration of 1 μmol/L.

### 3.4 Addition of LXR antagonists partially inhibited the mRNA levels of LXRs and ABC transporters compared to those treated with furanone alone

**Figure 4:**
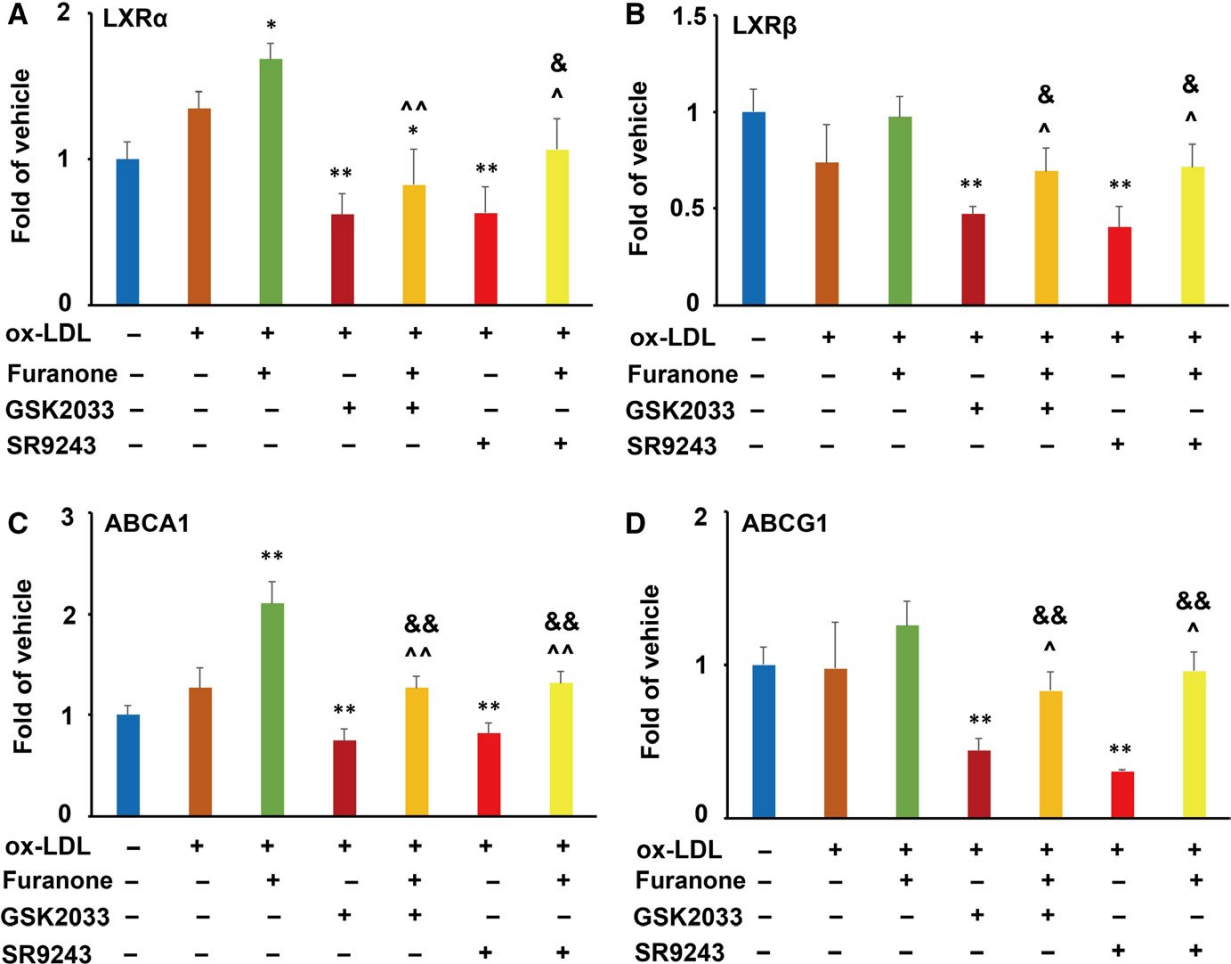
Effect of LXR antagonists GSK2033 and SR9243 on furanone‐induced mRNA expression of LXRα, LXRβ, ABCA1 and ABCG1 in RAW264.7 cells (n = 3). (A) Effect of LXR antagonists on the mRNA expression of LXRα induced by the furanone; (B) effect of LXR antagonists on the mRNA expression of LXRβ induced by the furanone; (C) effect of LXR antagonists on the mRNA expression of ABCA1 induced by the furanone; (D) effect of LXR antagonists on the mRNA expression of ABCG1 induced by the furanone. *means *P* < .05 vs model group; **means *P* < .01 vs model group; ^^^means *P* < .05 vs furanone; ^^^^means *P* < .01 vs furanone; ^&^means *P* < .05 vs antagonist; ^&&^means *P* < .01 vs antagonist

Further investigation using LXRs antagonists demonstrated that GSK2033 and SR9243 inhibited the mRNA levels of LXRα and LXRβ by ~52% and ~40%, respectively, (Figure 4A and B) compared to the model group. The furanone treatment significantly increased the mRNA levels of LXRα, but not LXRβ. Furthermore, the addition of LXRs antagonists partially inhibited the mRNA levels of LXRα (~50% for GSK2033 and ~30% for SR9243) and LXRβ (~28% for GSK2033 and SR9243) compared to the furanone alone treatment. That is to say, the addition of the furanone significantly increased the mRNA levels of LXRs compared with the treatment of LXR antagonists (Figure 4A and B). More importantly, LXR antagonist addition reduced the mRNA levels of ABCA1 and ABCG1 by ~43% and 36%, respectively, compared with the group treated with the furanone alone. On the other hand, compared to the antagonist alone treatment, the addition of this furanone significantly enhanced the mRNA levels of ABCA1 and ABCG1 by ~38% and 50%, respectively (Figure 4C and D).

### 3.5 The furanone lowered lipid levels in macrophages partially via enhancing the LXRα/ABC pathways

**Figure 5:**
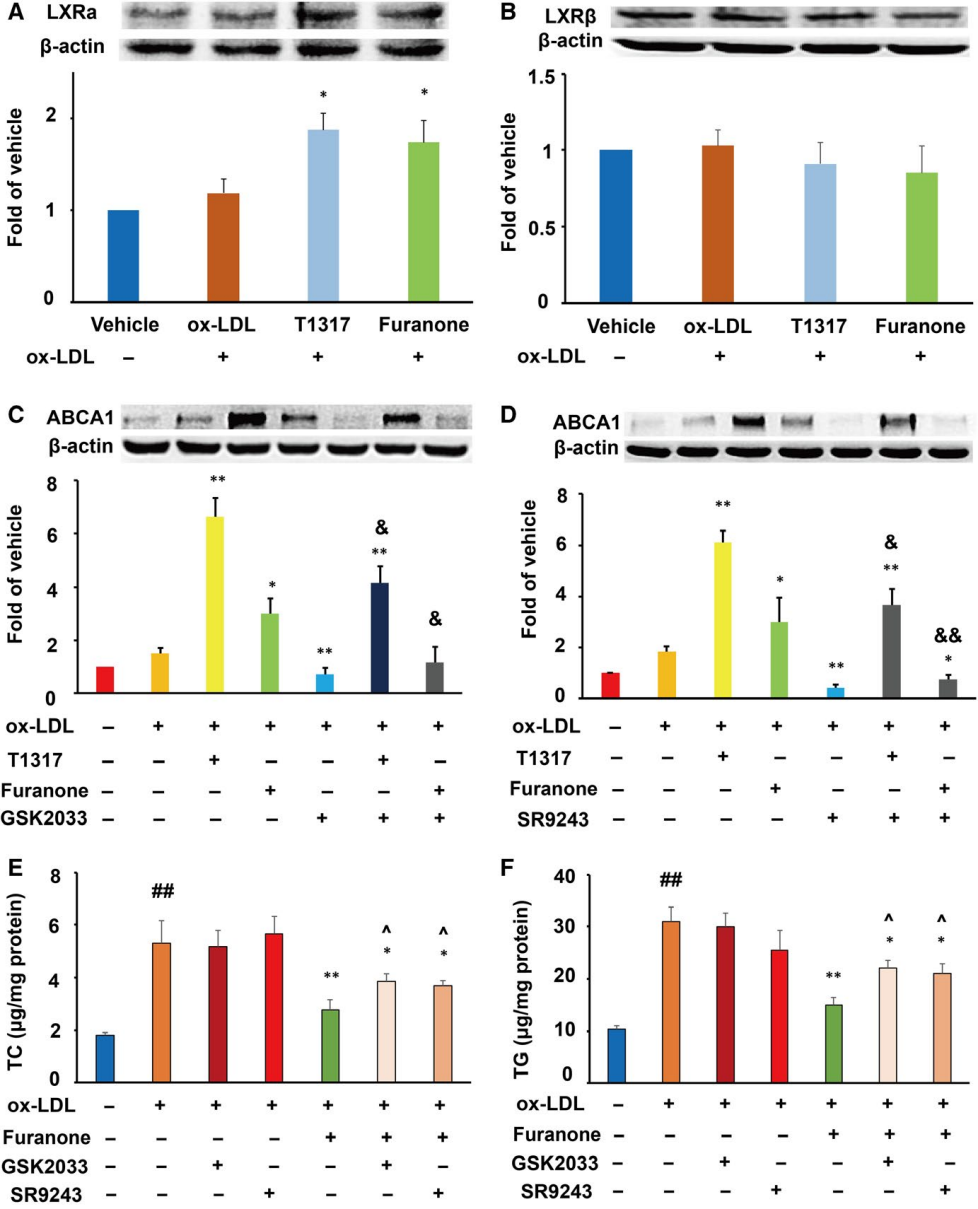
Effect of furanone on the protein expression of LXRs and the effect of LXR antagonists on furanone‐induced protein expression of ABCA1 and lipid accumulation in RAW264.7 macrophages. (A) Effect of the furanone on the protein expression of LXRα and densitometric quantification; (B) effect of the furanone on the protein expression of LXRβ and densitometric quantification; (C) effect of LXR antagonist GSK2033 on furanone‐induced protein expression of ABCA1; (D) effect of LXR antagonist SR9243 on furanone‐induced protein expression of ABCA1; (E) effect of LXR antagonists on furanone‐induced TC lowering; (F) effect of LXR antagonists on furanone‐induced TG lowering. Data are expressed as mean ± SD (n = 4). ^##^means *P* < .01 vs vehicle; *means *P* < .05 vs model group; **means *P* < .01 vs model group; ^&^means *P* < .05 vs the corresponding compound alone; ^&&^means *P* < .01 vs the corresponding compound alone; ^^^means *P* < .05 vs furanone treatment alone

As shown in Figure 5A and B, LXRα agonist T0901317 significantly improved the protein levels of LXRα, but not LXRβ compared with the model group. It was worth noting that the furanone, like T0901317, significantly enhanced the expression of LXRα but not LXRβ (Figure 5A and B). We also found that LXR antagonists GSK2033 and SR9243 inhibited the protein levels of ABCA1 by ~78% and 85% compared to the model group, respectively (Figure 5C and D). Furthermore, GSK2033 and SR9243 inhibited ~40% of the protein levels induced by the LXR agonist T0901317. More importantly, these two antagonists completely abolished the increase in protein levels of ABCA1 induced by the furanone. It was of note that the addition of LXR antagonists suppressed ~60% of the lipid‐lowering effect of the furanone (Figure 5E and F).

### 3.6 The TG‐lowering effect of the furanone was mainly attributed to its effect on PPARα in RAW264.7 cells

**Figure 6:**
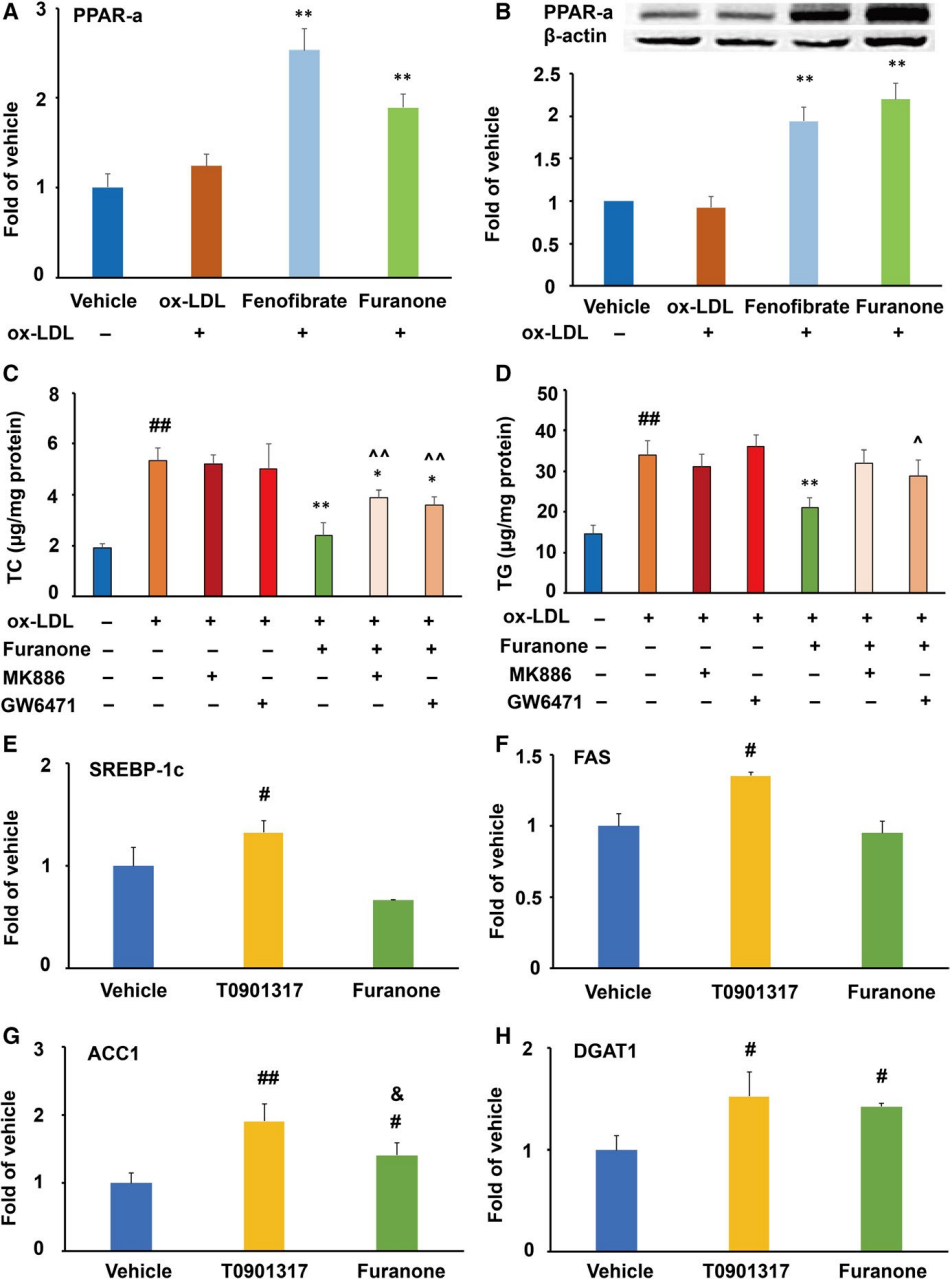
Effect of furanone on the expression of PPARα and TG synthesis‐related genes in RAW264.7 cells (n = 4). (A) mRNA expression of PPARα; (B) protein expression of PPARα densitometric quantification; (C) effect of PPARα antagonists on furanone‐induced TC lowering; (D) effect of PPARα antagonists on furanone‐induced TG lowering; (E) mRNA expression of SREBP‐1c; (F) mRNA expression of FAS; (G) mRNA expression of ACC1; H, mRNA expression of DGAT1. ^#^means *P* < .05 vs vehicle; ^#^means *P* < .05 vs vehicle; ^##^means *P* < .01 vs vehicle; *means *P* < .05 vs model group; **means *P* < .01 vs model group; ^^^means *P* < .05 vs furanone treatment alone; ^^^^means *P* < .01 vs antagonist treatment alone; ^&^means *P* < .05 vs T0901317

Compared to the model group, PPARα agonist fenofibrate significantly improved the mRNA and protein levels of PPARα by ~2.5‐fold and ~1.9‐fold, respectively (*P* < .01, Figure 6A and B). Furthermore, the furanone increased the mRNA and protein levels of PPARα by ~48% and 130%, respectively, compared to the model group (*P* < .01). It was of note that PPARα antagonists MK886 and GW6471 reduced the TC‐ and TG‐lowering effect of the furanone from 55% to 34% and from ~40% to ~12%, respectively (Figure 6C and D). These data suggest that PPAR antagonists reduce TC‐ and TG‐lowering effect of the furanone by ~38% and 70%, respectively, and indicate that the PPARα pathway plays an important role in the lipid‐lowering effect of the furanone in RAW264.7 cells.

To further understand why the furanone had a better TG reduction effect than T0901317, we measured the mRNA levels of genes involved in fatty acid biosynthesis, elongation and desaturation via RT‐PCR. As shown in Figure 6E‐H, LXRα agonist T0901317 significantly enhanced the mRNA levels of SREBP‐1c, FAS, ACC1 and DGAT1 compared to the vehicle (*P* < .01 or *P* < .05). Although the furanone significantly increased the mRNA levels of ACC1 and DGAT1 by ~40% (*P* < .05), but not as much as T0901317, especially for that of ACC1 (Figure 6G, *P* < .05 compared to the T0901317‐treated group). Additionally, the mRNA levels of SCD1 and DGAT2 were undetectable; Ct numbers were ~30.

### 3.7 The furanone reduced lipid levels in oleic acid‐loaded HepG2 cells

**Figure 7:**
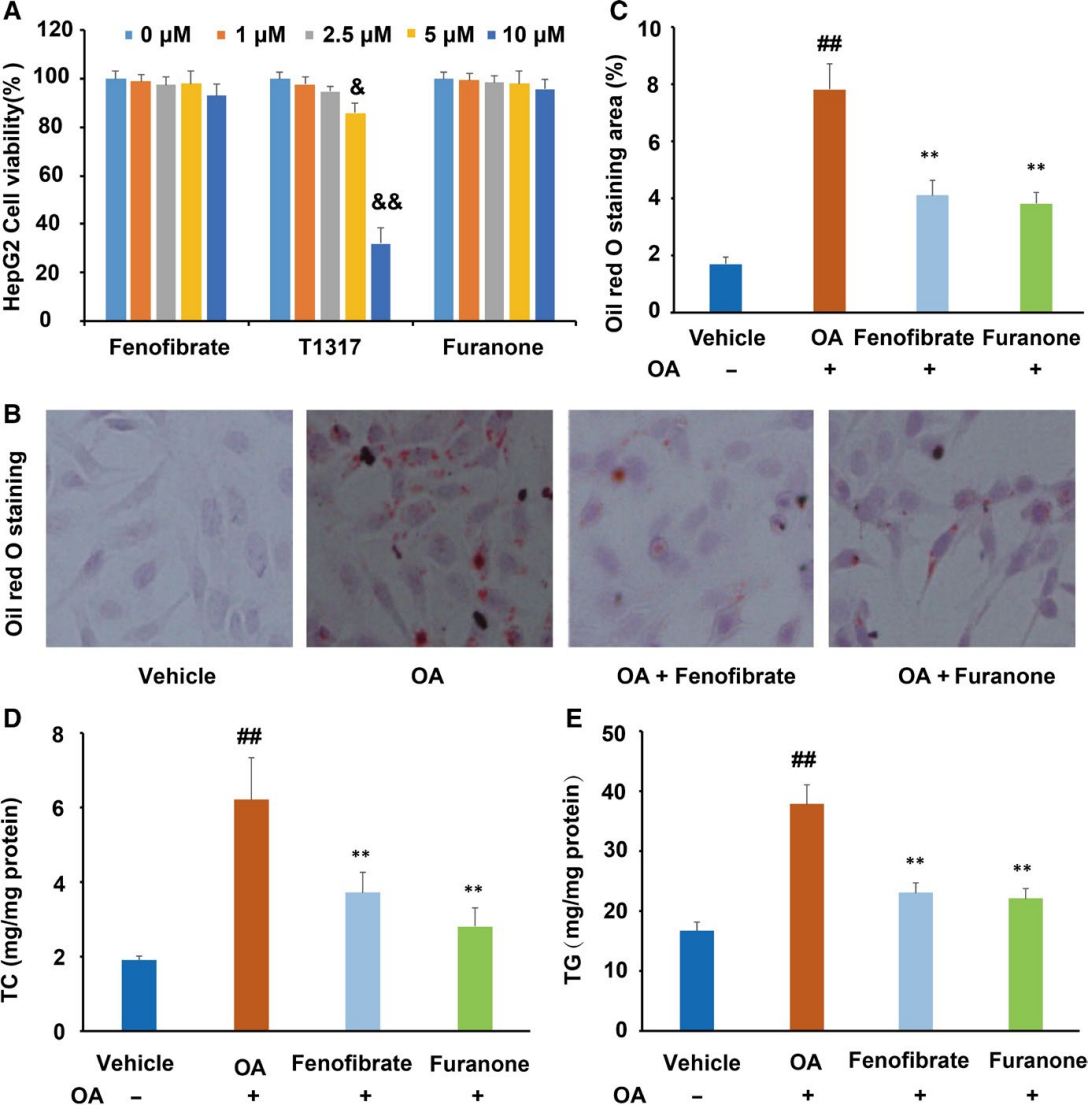
Cytotoxicity and lipid‐lowering effect of the furanone in HepG2 cells. (A) Viability of HepG2 cells in the presence of 0‐10 μmol/L furanone, fenofibrate or liver X receptor agonist T0901317; (B) typical pictures of Oil Red O staining (100 ×); (C) percentage of Oil Red O staining area in HepG2 cells; (D) intracellular levels of total cholesterol (TC); (E) intracellular levels of triglyceride (TG). Data are expressed as mean ± SD (n = 4). OA: oleic acid. ^&^means *P* < .05 vs 0 μmol/L; ^&&^means *P* < .01 vs μmol/L; ^##^means *P* < .01 vs vehicle; **means *P* < .01 vs model group; ^&^means *P* < .05 vs fenofibrate group

The cytotoxicity effect of the furanone on HepG2 cells was similar to that on the RAW264.7 cells (Figure 7A). Although palmitic acid alone or in combination with oleic acid could induce lipid accumulation, palmitic acid is not as effective as oleic acid and palmitic acid can induce the expression of PPARα.^34^ Oleic acid alone is generally used to induce lipid accumulation in HepG2 cells.^35^ Therefore, oleic acid was used in the present study. Oil Red O staining results demonstrated that 0.5 mmol/L oleic acid treatment significantly increased lipid accumulation in HepG2 cells (~3.3‐fold, *P* < .01, Figure 7B and C), and fenofibrate at 5 μmol/L can markedly decrease the cellular lipid levels compared to the model group (Figure 7B and C). Further assays indicated that fenofibrate significantly decreased TC (~31.5%, Figure 7D, *P* < .01) and TG (~47.4%, Figure 7E, *P* < .01) levels in HepG2 cells when compared to the model group. Interestingly, the furanone also significantly reduced the levels of TC (~78.2%, *P* < .01, Figure 7D) and TG (~48.7%, *P* < .01, Figure 7E) in HepG2 cells when compared with the model group.

### 3.8 The furanone enhanced LDLR and decreased SREBP‐2 expression in HepG2 cells

**Figure 8:**
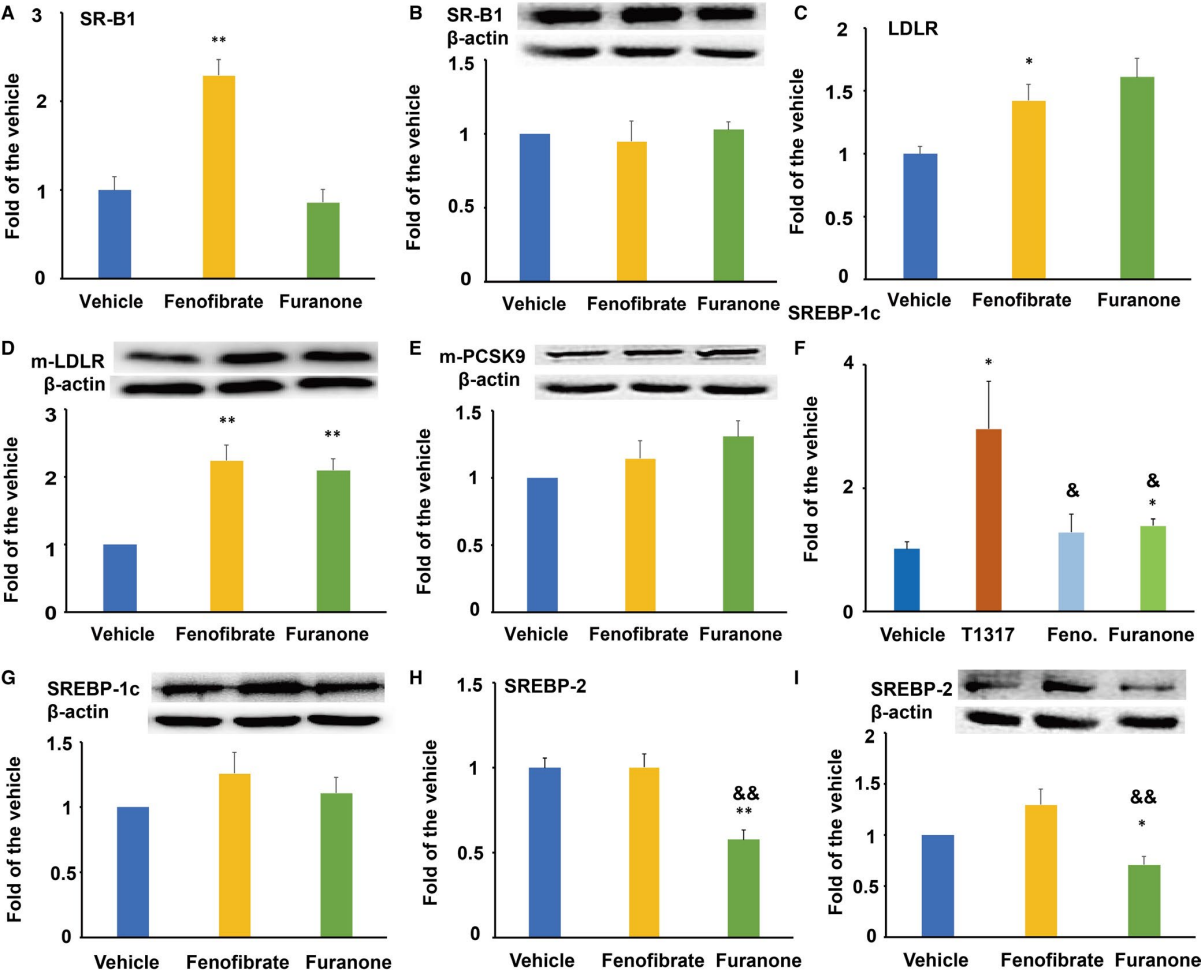
Effect of the furanone on the expression of SR‐B1, LDLR, PCSK9 and SREBPs in HepG2 cells. (A) mRNA expression of SR‐B1; (B) protein expression of SR‐B1 and densitometric quantification; (C) mRNA expression of LDLR; (D) protein expression of LDLR and densitometric quantification; (E) protein expression of PCSK9 and densitometric quantification. (F) mRNA expression of SREBP‐1c; (G) protein expression of SREBP‐1c and densitometric quantification; (H) mRNA expression of SREBP‐2; I, protein expression of SREBP‐2 and densitometric quantification. Data are expressed as mean ± SD (n = 4). *means *P* < .05 vs vehicle; **means *P* < .01 vs vehicle; ^&^means *P* < .05 vs fenofibrate group; ^&&^means *P* < .01 vs fenofibrate group

SR‐B1 and LDLR mediate the transfer of HDL cholesterol and non‐HDL lipids from plasma to the liver for metabolism, respectively. In this study, we found that the furanone showed no significant effect on the mRNA and protein levels of SR‐B1 (Figure 8A and B). However, like fenofibrate, it significantly increased the mRNA (~1.5‐fold) and protein (~2.1‐fold) levels of LDLR as that of fenofibrate (Figure 8C and D, *P* < .01). Furthermore, both fenofibrate and the furanone showed no significant effect on the protein levels of PCSK9 (Figure 8E). Additionally, fenofibrate significantly increased SR‐B1 mRNA but not protein levels.

SREBPs are important transcription factors involved in the regulation of lipid metabolism and homeostasis in the liver. As shown in Figure 8F‐I, fenofibrate treatment showed no significant effect on the mRNA and protein levels of SREBP‐1c and SREBP‐2. In this study, the furanone significantly enhanced the mRNA levels of SREBP‐1c (~27%, *P* < .05, Figure 8F) without any effect on the protein levels (Figure 8G). Furthermore, the effect of the furanone on the mRNA levels of SREBP‐1c was similar to that of fenofibrate (Figure 8F), but to a much lesser extent than T0901317 (*P* < .05). It was worth noting that this furanone significantly reduced the mRNA (~35%, *P* < .01, Figure 8H) and protein levels of SREBP‐2 (~29%, *P* < .05, Figure 8I). Furthermore, the furanone exhibited a significant difference in the inhibitory effect of SREBP‐2 compared to fenofibrate (~50%, *P* < .01, Figure 8H and I).

### 3.9 The furanone increased the expression of ABCG5 and ABCG8 in HepG2 cells

**Figure 9:**
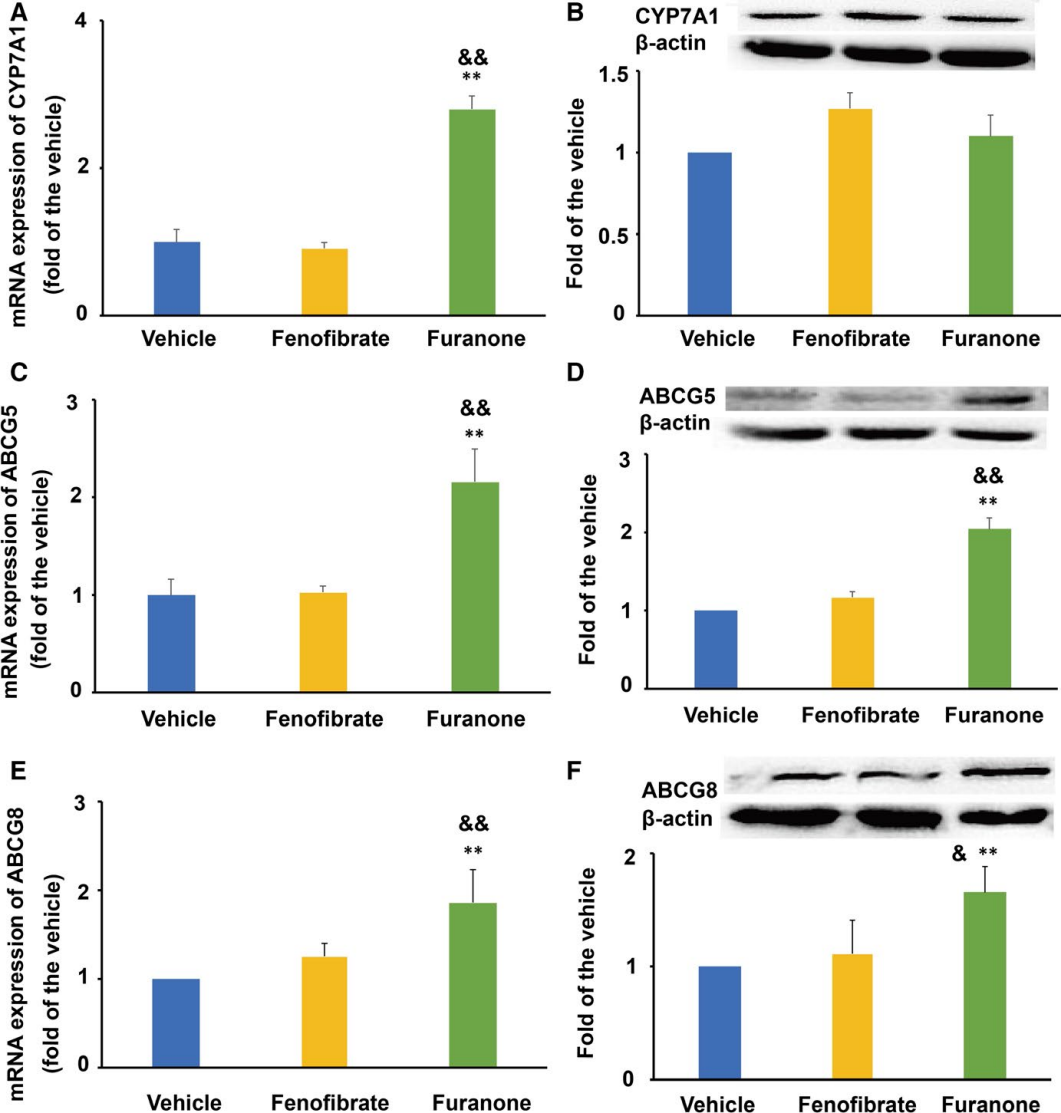
Effect of the furanone on the expression of CYP7A1, ABCG5 and ABCG8 in HepG2 cells. (A) mRNA expression of CYP7A1; (B) protein expression of CYP7A1 and densitometric quantification; (C) mRNA expression of ABCG5; (D) protein expression of ABCG5 and densitometric quantification; (E) mRNA expression of ABCG8; (F) protein expression of ABCG8 and densitometric quantification. Data are expressed as mean ± SD (n = 4). **means *P* < .01 vs vehicle; ^&^means *P* < .05 vs fenofibrate group; ^&&^means *P* < .01 vs fenofibrate group

CYP7A1 is an important rate‐limiting enzyme in the biosynthesis process of bile acids. In this study, we found that fenofibrate did not affect the mRNA and protein levels of CYP7A1 (Figure 9A and B). On the other hand, the furanone significantly elevated the mRNA levels of CYP7A1 compared to the model or fenofibrate group (~3‐fold, Figure 9A, *P* < .01), but did not affect the protein levels of CYP7A1 (Figure 9B). It was of note that the furanone significantly elevated the mRNA and protein levels of ABCG5 (Figure 9C and D, ~2‐fold, *P* < .01) and ABCG8 (Figure 9E and F, ~1.8‐fold) compared to the model group. The effects of the furanone on ABCG5 and ABCG8 were stronger than that of fenofibrate that showed no significant effects on the expression of either ABCG5 or ABCG8 (Figure 9C‐F, *P* < .01 or *P* < .05).

### 3.10 The furanone lowered lipid by modulating PPARα in HepG2 cells

**Figure 10:**
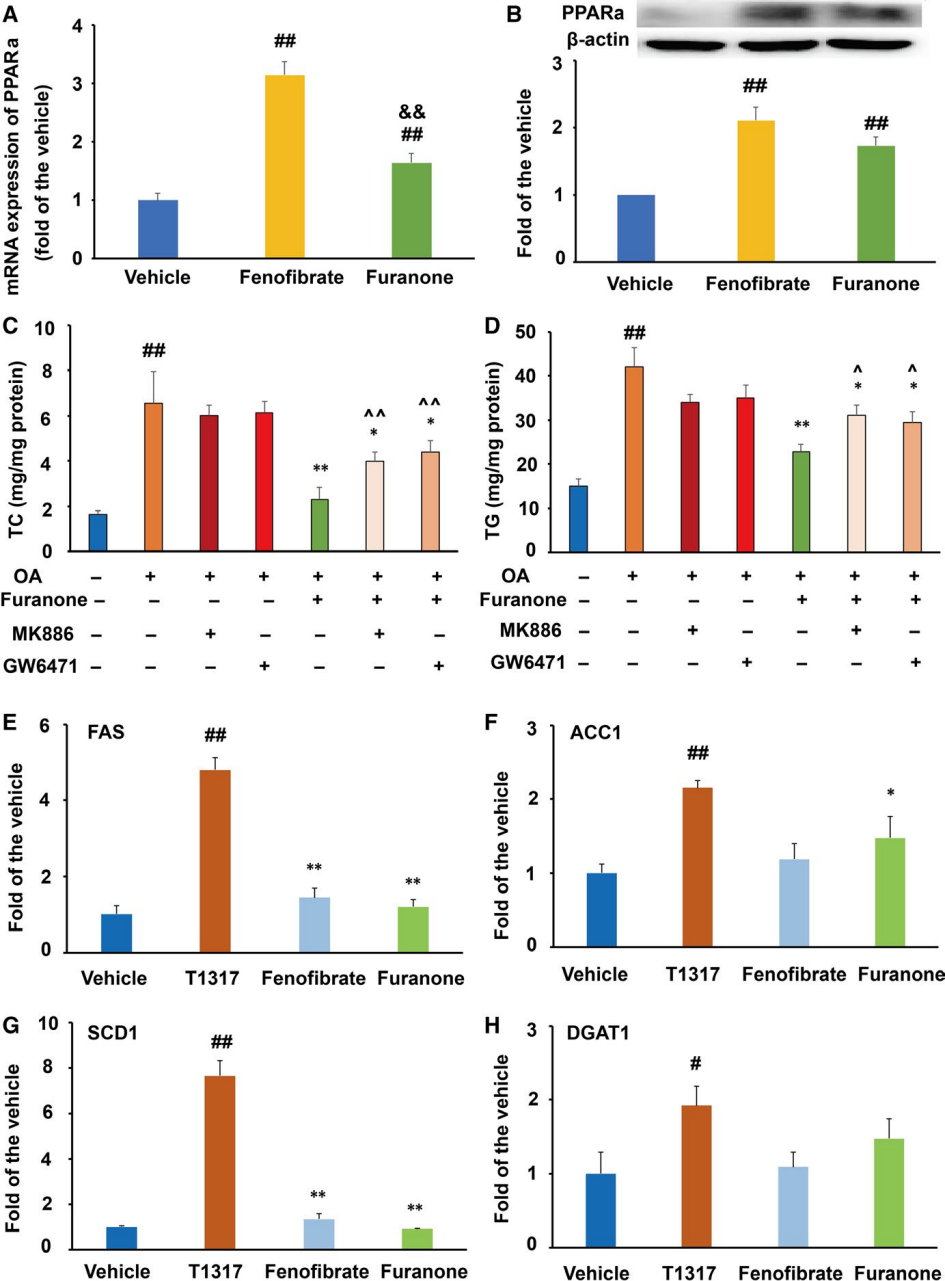
This furanone lowered lipids by enhancing PPARα in HepG2 cells. (A) mRNA expression of PPARα; (B) protein expression of PPARα and densitometric quantification; (C) PPARα antagonists reduced the TC‐lowering effect of the furanone; (D) PPARα antagonists significantly reduced the TG‐lowering effect of the furanone; (E) mRNA expression of FAS; (F) mRNA expression of ACC1; (G) mRNA expression of SCD1; H, mRNA expression of DGAT1. Data are expressed as mean ± SD (n = 3). ^#^means *P* < .05 vs vehicle; ^##^means *P* < .01 vs vehicle; ^&&^means *P* < .01 vs fenofibrate. In Fig. C and D, *means *P* < .05 vs OA alone; **means *P* < .01 vs OA alone; ^^^means *P* < .05 vs furanone (without antagonist) group; ^^^^means *P* < .01 vs furanone (without antagonist) group. In Fig. E‐H, *means *P* < .05 vs T0901317; **means *P* < .01 vs T0901317

The furanone significantly improved the mRNA (~1.6‐fold, *P* < .05) and protein (~1.8‐fold, *P* < .01) levels of PPARα (Figure 10A and B). Although the effect of the furanone on the mRNA levels of PPARα was weaker than that of fenofibrate (Figure 10C, *P* < .01), there was no significant difference in the protein levels between the two groups (Figure 10B). To define the role of PPARα in the anti‐hyperlipidaemic effect of the furanone, two antagonists were used in the following study. As shown in Figure 10C and D, the TC‐lowering effect of the furanone was changed from 63% (without antagonist) to 35% (with antagonist, Figure 10C, *P* < .01), and the TG‐lowering effect was changed from 45% (without MK886) to 16.0% (with MK886, Figure 9D, *P* < .05). These results indicated that PPARα antagonists inhibited the TC‐ and TG‐lowering effect of the furanone by ~44% and 64%, respectively. Therefore, the TG‐lowering effect of the furanone was mainly attributed to its up‐regulation of PPARα in HepG2 cells.

To further investigate the TG‐lowering effect of the furanone, the mRNA levels of genes involved in fatty acid biosynthesis, elongation and desaturation were investigated using RT‐PCR. As shown in Figure 10E‐H, LXRα agonist T0901317 significantly enhanced the mRNA levels of FAS, ACC1, SCD1 and DGAT1 compared to that of the vehicle (*P* < .01 or *P* < .05). The furanone promoted the mRNA levels of ACC1 (~47% increase, *P* < .05); however, the effect was lower than that of T0901317 (Figure 10F). It was of note that the effects of the furanone on the mRNA levels of FAS and SCD1 were significantly lower compared to that of T0901317 (Figure 10E and G, *P* < .01). Additionally, the mRNA levels of DGAT2 were undetectable.

## 4 DISCUSSION

RCT is a physiological process in which excess peripheral cholesterol is transported to the liver for excretion into the bile and then faeces.^22^ It is believed that RCT participates in the reduction of hyperlipidaemia and cardiovascular disease.^36^, ^37^ To imitate the RCT processes in vivo, we investigated the lipid‐lowering effect of the furanone in different cell lines. RAW264.7 macrophages and HepG2 cells represent the peripheral cells and liver cells, respectively.

During the first step of RCT, ABCA1 mediates cholesterol efflux from peripheral cells to apolipoprotein (apo) A1 and pre‐β HDL; and ABCG1 and SR‐B1 mediate cholesterol efflux from peripheral cells to mature HDL.^22^, ^36^, ^37^ Our data indicated that the furanone may enhance the first step of RCT by increasing the expression of ABCA1, ABCG1 and SR‐B1. LXRα has been demonstrated to be an activator of ABC transporters and SR‐B1.^38^ Thus, we proposed that LXRα might play an important role in the furanone‐induced expression of ABC transporters. Indeed, LXR antagonists, GSK2033 and SR9243, partially suppressed the expression of ABCA1 and ABCG1 and the anti‐hyperlipidaemic effect induced by the furanone in lipid‐laden RAW264.7 macrophages. Taken together, the furanone may enhance cholesterol efflux from peripheral cells to the circulation by up‐regulation of the LXR/ABC pathways. LXR antagonists GSK2033 and SR9243 efficiently decreased the expression of LXRα and its target genes, such as ABCA1, and abolished the effects of LXR agonist T0901317 (Figure 4). However, GSK2033 and SR9243 had no effect on cellular levels of lipids (Figure 5), consistent with previous studies.^39^, ^40^, ^41^ For example, although GSK2033 significantly reduced the protein levels of LXRα, it showed no significant effect on lipid accumulation in apolipoprotein E‐deficient mice and foam cell formation of RAW264.7 cells compared to the model group.^39^ Furthermore, SR9243 showed no effect on the TG accumulation in zebrafish hepatocytes.^40^ These promiscuous activities of the LXR antagonists may be due to their unexpected effects on lipogenic genes. For instance, GSK2033 could significantly improve rather than suppress the expression of the lipogenic genes such as FAS and SREBP‐1c and therefore had no effect on hepatic steatosis in a mouse model of non‐alcoholic fatty liver disease.^41^ Furthermore, these antagonists may target a number of other nuclear receptors, such as the glucocorticoid receptor, pregnane X receptor and farnesoid X receptor, all of which can definitely alter hepatic gene expression.^41^ However, the underlying mechanisms of these antagonists on lipid metabolism need to be further investigated.

The liver is an important organ for the second step of RCT and lipid metabolism. LDLR and SR‐B1 mediate the transfer of non‐HDL cholesteryl ester and HDL cholesterol to the liver for metabolism, respectively.^22^, ^36^, ^37^ The results of fenofibrate on LDLR and SR‐B1 were consistent with previous reports.^22^, ^42^ Here, we demonstrated that this furanone might enhance the transfer of cholesteryl ester from plasma to the liver by elevating LDLR expression as that of fenofibrate.^38^ PCSK9 binds to the epidermal growth factor‐like repeat A domain of the LDLR, inducing LDLR degradation.^43^ However, the elevated LDLR level induced by this furanone was unrelated to PCSK9. Although fenofibrate was reported to down‐regulate the PCSK9 expression in diabetic patients,^44^ our data indicated that fenofibrate had no effect on PCSK9 in HepG2 cells, and the result was consistent with a previous report.^45^ Hepatic cholesterol transported to the liver can be converted to 7‐alpha‐hydroxycholesterol by CYP7A1, the rate‐limiting enzyme in bile acid synthesis.^22^ Our data indicated that the furanone might have no influence on the conversion of cholesterol to bile acids. However, this furanone may accelerate lipid secretion in HepG2 cells by improving the expression of ABCG5 and ABCG8. Additionally, the effects of fenofibrate on CYP7A1 and ABCG5 were consistent with previous publications.^46^, ^47^ The inconsistence of the effects on the mRNA and protein levels of SR‐B1, SREBP‐1c (Figure 8) and CYP7A1 (Figure 9) may be attributed to the unknown post‐transcriptional modifications.

SREBPs are important transcription factors involved in the regulation of lipid metabolism and homeostasis in the liver.^48^ SREBP‐1c regulates the expression of lipogenic genes, such as ACC and FAS, thereby regulating the fatty acid and TG synthesis. During the process of TG synthesis, acetyl‐CoA is carboxylated to malonyl‐CoA by ACC. FAS catalyses malonyl‐CoA into palmitate. SCD1 catalyses the synthesis of monounsaturated fatty acids, which are then incorporated into TGs and phospholipids; DGATs are also important to synthesize fatty acids into TGs.^18^, ^40^ The elevated levels of these genes indicated that T0901317 treatment might increase the de novo lipogenesis in RAW264.7 cells and HepG2 cells, which were consistent with previous studies.^49^, ^50^ The low expression of these genes in the furanone‐treated cells may partially explain the better TG‐lowering effect of the furanone compared to T0901317 in RAW264.7 cells. SREBP‐2 specifically activates the transcription of genes involved in cholesterol metabolism, such as HMG‐CoA reductase and LDLR, thereby regulating cholesterol biosynthesis.^48^ The results of fenofibrate on SREBPs were consistent with previous reports.^51^, ^52^ Our data demonstrated that the lipid‐lowering effect of this furanone may be partially attributed to the down‐regulation of SREBP‐2 in HepG2 cells. Fenofibrate did not significantly modulate the expression of SREBP‐2 (Figure 8I) in the present study, which was consistent with previous reports.^51^, ^52^

Furthermore, PPARα agonists, fibrates, have been successfully explored as anti‐hyperlipidaemic drugs, especially for TG lowering.^53^, ^54^ We demonstrated that the furanone, like fenofibrate, increased the protein and mRNA levels of PPARα in both RAW264.7 and HepG2 cells. Interestingly, PPARα antagonist MK886 and GW6471 could only partially inhibit the lipid‐lowering effect of the furanone. However, its anti‐hyperlipidaemic effects could not be fully abolished. Therefore, the furanone may exert its lipid‐lowering effect, especially on TG, via up‐regulating PPARα in RAW 264.7 and HepG2 cells as that of fenofibrate,^47^, ^54^ but other unknown mechanisms also contribute to this effect. In addition, the PPARα inhibitors, MK886 and GW6471, had no effects on cellular lipid levels in HepG2 cells. Consistently, it has been reported that MK886 had no significant effect on oleic acid‐induced lipid accumulation in cells.^30^ The underlying mechanisms may be similar to those of LXR antagonists 41 as aforementioned.

Hundreds of furanone derivatives have been synthesized during the past decades, and these compounds exhibited various activities.^19^

Based on our findings, the furanone may not induce severe lipid accumulation in the liver as those of LXR agonists due to its weak effect on the expression of TG biogenesis genes. However, the lipid‐lowering mechanisms of the furanone need to be further investigated in other models and especially in vivo models. We will synthesize a series of the furanone‐based compounds and then investigate the association between their structure and activity.

## CONFLICT OF INTEREST

The authors declare that there are no conflicts of interest in this manuscript.

## AUTHOR CONTRIBUTIONS

XP, JW and YL separated and provided the compound. SH, TL, JY, JW, XY and BX conducted the experiments. FL, WS and SG contributed reagents and the design of this study. SH, TL and SG performed the data analysis and contributed to the writing of the manuscript.

## Data Availability

Data are available on requirement.
