# Correction to “The marine‐derived furanone reduces intracellular lipid accumulation in vitro by targeting LXRα and PPARα”

**DOI:** 10.1111/jcmm.70108

**Published:** 2024-11-01

Li T, Hu SM, Pang XY, et al. The marine‐derived furanone reduces intracellular lipid accumulation in vitro by targeting LXRα and PPARα. *J Cell Mol Med*. 2020;24:3384‐3398. doi:10.1111/JCMM.15012

In Ting Li et al., several incorrect images were used in Figure 3B. The correct figure is shown below. The related fluorescent images were shown in the Supporting Information. The authors confirm all results and conclusions of this article remain unchanged.

**FIGURE 3:**
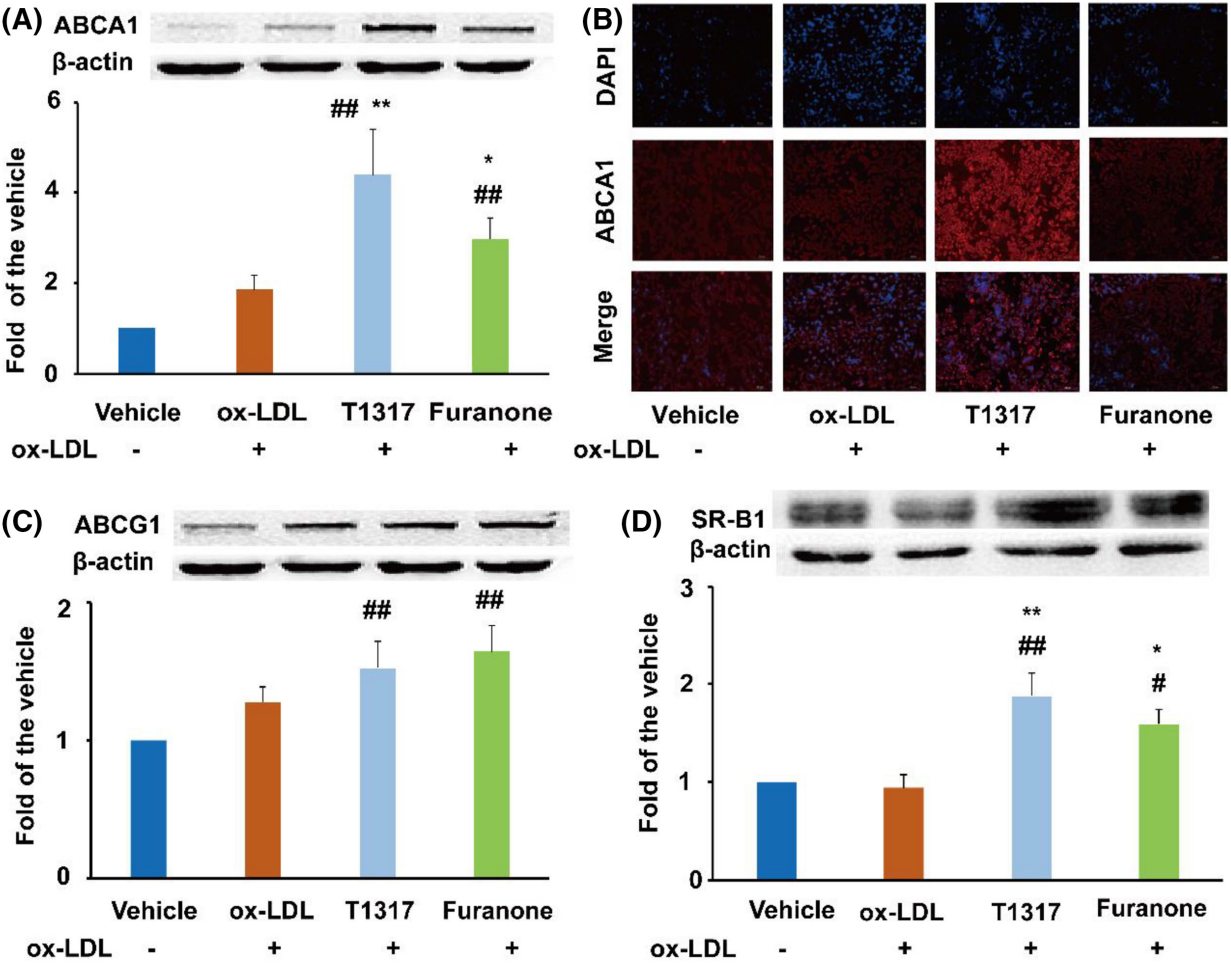
Effect of the furanone on enhancing the protein expression of ABCA1, ABCG1 and SR‐B1 in RAW264.7 cells. (A) Protein expression of ABCA1 and densitometric quantification; (B) detection of ABCA1 expression by fluorescent immunocytochemistry; (C) protein expression of ABCG1 and densitometric quantification; (D) protein expression of SR‐B1 and densitometric quantification. Data are expressed as mean ± SD (*n* = 4). ^#^means *p* < 0.05 vs. vehicle; ^##^means *p* < 0.01 vs. vehicle; *means *p* < 0.05 vs. model group; **means *p* < 0.01 vs. model group.

## Supporting information


Data S1.

