# Supplementary material for: Correction to “The marine‐derived furanone reduces intracellular lipid accumulation in vitro by targeting LXRα and PPARα”
Source: J Cell Mol Med. 2024 Nov 1;28(21):e70108. doi: 10.1111/jcmm.70108 (PMC11528241; doi:10.1111/jcmm.70108)
Supplement: Supplementary file 1 — Data S1. [file JCMM-28-e70108-s001.pdf]

Figure 3B Fluorescent images (Raw data)

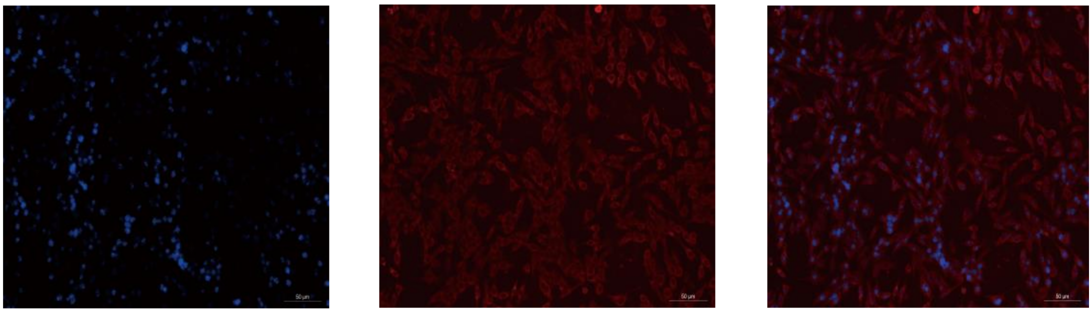

Vehicle: DAPI ABCA1 Merge

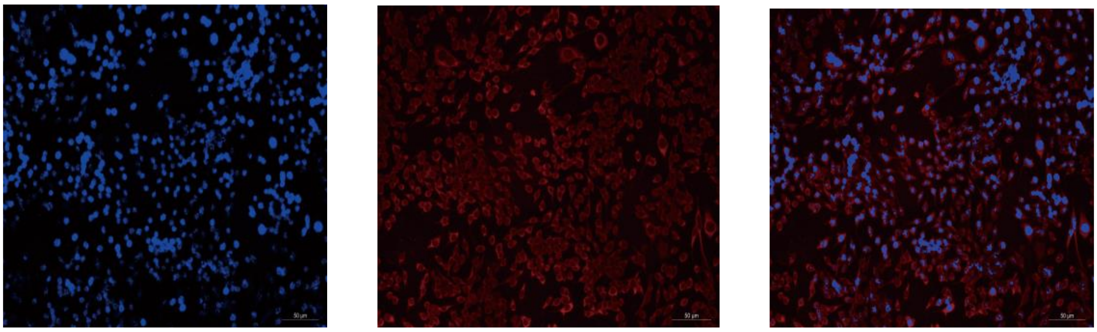

Ox-LDL: DAPI ABCA1 Merge

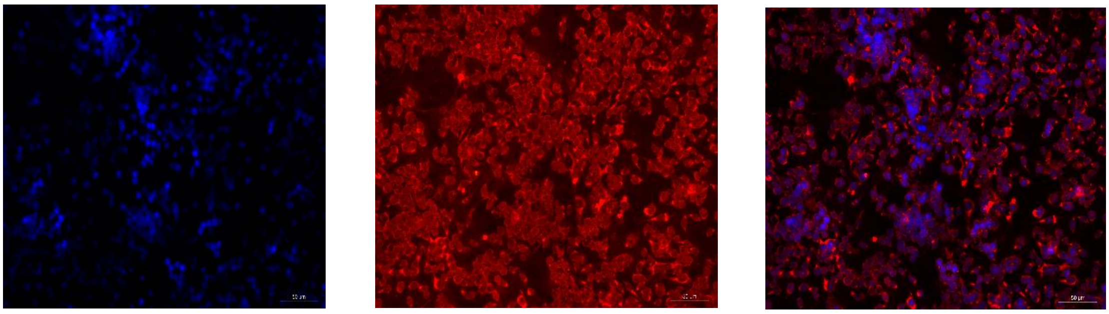

T1317: DAPI ABCA1 Merge

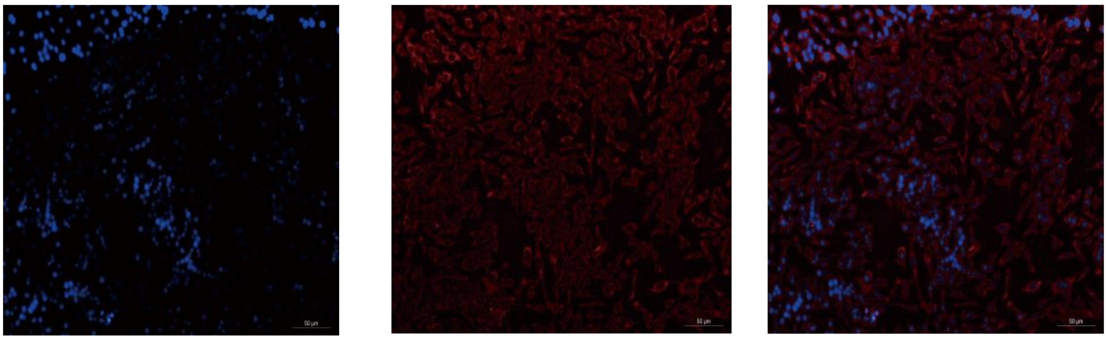

Furanone: DAPI ABCA1 Merge
